# Characterizing and utilizing oxygen-dependent promoters for efficient dynamic metabolic engineering

**DOI:** 10.1016/j.ymben.2023.04.006

**Published:** 2023-05

**Authors:** Julian Wichmann, Gerrich Behrendt, Simon Boecker, Steffen Klamt

**Affiliations:** Analysis and Redesign of Biological Networks, Max Planck Institute for Dynamics of Complex Technical Systems, Sandtorstr. 1, 39106, Magdeburg, Germany

**Keywords:** Oxygen-responsive promoters, *Escherichia coli*, Two-stage processes, Dynamic metabolic engineering, Bioprocess design, Enforced ATP wasting, Lactate

## Abstract

Promoters adjust cellular gene expression in response to internal or external signals and are key elements for implementing dynamic metabolic engineering concepts in fermentation processes. One useful signal is the dissolved oxygen content of the culture medium, since production phases often proceed in anaerobic conditions. Although several oxygen-dependent promoters have been described, a comprehensive and comparative study is missing. The goal of this work is to systematically test and characterize 15 promoter candidates that have been previously reported to be induced upon oxygen depletion in *Escherichia coli*. For this purpose, we developed a microtiter plate-level screening using an algal oxygen-independent flavin-based fluorescent protein and additionally employed flow cytometry analysis for verification. Various expression levels and dynamic ranges could be observed, and six promoters (nar-strong, nar-medium, nar-weak, nirB-m, yfiD-m, and fnrF8) appear particularly suited for dynamic metabolic engineering applications. We demonstrate applicability of these candidates for dynamic induction of enforced ATP wasting, a metabolic engineering approach to increase productivity of microbial strains that requires a narrow level of ATPase expression for optimal function. The selected candidates exhibited sufficient tightness under aerobic conditions while, under complete anaerobiosis, driving expression of the cytosolic F_1_-subunit of the ATPase from *E. coli* to levels that resulted in unprecedented specific glucose uptake rates. We finally utilized the nirB-m promoter to demonstrate the optimization of a two-stage lactate production process by dynamically enforcing ATP wasting, which is automatically turned on in the anaerobic (growth-arrested) production phase to boost the volumetric productivity. Our results are valuable for implementing metabolic control and bioprocess design concepts that use oxygen as signal for regulation and induction.

## Highlights

• 15 oxygen-dependent promoters systematically characterized.
• Microtiter plate level screening using an oxygen-independent LOV fluorescent reporter.
• Verification of expression levels and dynamic ranges using flow cytometry.
• Six most suitable promoters for dynamic metabolic engineering applications identified.
• Example study: nirB-m driven ATP wasting boosts lactate productivity in a two-stage process.

## Graphical abstract

**Figure:**
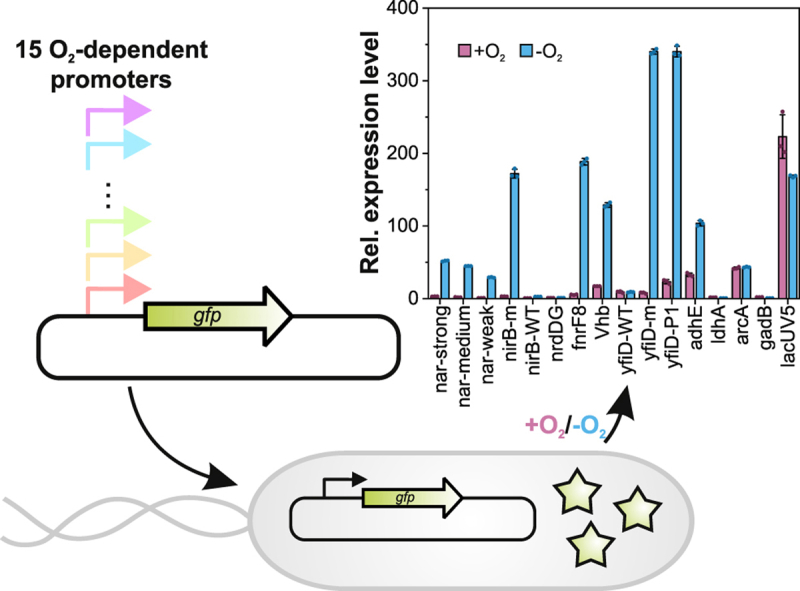

## 1 Introduction

Synthetic biology tools have significantly accelerated the implementation of metabolic engineering strategies to develop and expand microbial production concepts towards new substrates, products, and hosts with the ultimate goal to maximize the cost-efficiency of bioprocesses. Promoters are key targets for this purpose as they represent the first level of genetic control over enzyme abundances and thus regulate, for example, the activity of biosynthetic pathways (Keasling, 2012). Different constitutive promoters can be used to adjust the strength of expression of a certain gene. Another class is inducible promoters, which have an additional degree of control and can be used to switch on a gene when its expression is needed, e.g. in the production phase of a two-stage process. There are various application examples of inducible promoters and they have also been engineered (or even synthetically been constructed) to fine-tune their strength and dynamic range or to change the input signal (Blazeck and Alper, 2013; Cazier and Blazeck, 2021; Liu et al., 2019; Xu et al., 2019). Frequently used inducers are small molecules such as arabinose or IPTG. However, due to interference of arabinose with catabolite repression (Miyada et al., 1984) and the relatively high price of IPTG, their use is often economically impractical for large-scale production of bulk products. Alternatively, external signals (pH, temperature, nutrients, oxygen, products) or cellular factors (growth phase, metabolites, redox state) can be leveraged with suitable genetically encoded sensors to dynamically modulate transcription (Moser et al., 2018). Basic principles and applications of these systems have been reviewed extensively elsewhere (Hartline et al., 2021; Lalwani et al., 2018; Venayak et al., 2015), and recent engineering examples include the use of cell density (Gupta et al., 2017), temperature (Harder et al., 2018), phosphate (Menacho-Melgar et al., 2020), and oxygen tension (Hwang et al., 2017) as signals to achieve dynamic genetic and metabolic control. Two-stage microbial production processes, in which cell growth is separated from the production phase, represent an attractive opportunity to put these systems to use: in a first stage, biomass is accumulated and then, at the beginning of the second (production) phase, triggered to become a bio-catalyst by switching relevant genes on or off by which most of the carbon taken up is diverted into the desired product(s). Compared to one-stage processes with simultaneous production of biomass and product, this may boost titers, rates, and yields, the key performance indicators of large-scale bioprocesses (Burg et al., 2016).

Oxygen-dependent promoters may be of high value for two-stage processes in which an aerobic growth phase is followed by an anaerobic production phase. Many oxygen-dependent promoters are regulated by the fumarate and nitrate reductase (FNR) operator. Its transcriptional activator FNR is a global transcriptional regulator of many genes relevant in anaerobic metabolism (Constantinidou et al., 2006; Sawers et al., 1988) and contains an Fe–S cluster which is modified by oxygen. When active in the absence of oxygen, a [4Fe–4S]^2+^ cluster is stable, causing dimerization of FNR and binding to its operator, a state which is abolished in the presence of oxygen (Kiley and Beinert, 1998). FNR also acts as a repressor of genes whose expression is required under aerobic conditions (Guest et al., 1996). Several oxygen-dependent promoter candidates have been presented individually in the past, including synthetic candidates and promoters with randomized spacer sequences or few substituted base pairs (Table 1). However, a systematic and comprehensive comparison of these candidate promoters is lacking and there are still only few reported applications (Hwang et al., 2017, 2018; Mehrer et al., 2018) making use of oxygen-responsive promoters for metabolic control and process design. This is surprising since two-stage processes with an aerobic growth and an anaerobic production phase seem relevant for many production scenarios.

**Table 1:** Overview of oxygen-dependent promoters investigated in this study. *gadB was found to be strongly upregulated in microarray data of GenExpDB, accession GSE4375.

Promoter name	References	Engineering strategies applied previously	Size (bp) Until TSS	Regulator
nar (synthetic versions):	(Walker and DeMoss, 1992), (Hwang et al., 2018)	Randomization of spacer region to modulate strength	54	FNR
(1) nar-strong				
(2) nar-medium				
(3) nar-weak				
nirB	Oxer et al. (1991)	Deletion of nitrite-responsive part (nirB-WT, nirB-m), incorporation of FNR consensus sequence (nirB-m)	58	FNR
(4) nirB-WT				
(5) nirB-m				
(6) nrdDG	Roca et al. (2008)	–	87	FNR
(7) fnrF8	Moser et al. (2018)	Synthetic: placement of consensus FNR operator into constitutive promoters, optimization by randomizing spacer sequences using oligonucleotide arrays & FACS	51	FNR
(8) VHb	(Dikshit et al., 1990; Khosla et al., 1990; Khosla and Bailey, 1989; Mehrer et al., 2018)	–	86	FNR
yfiD	(Green et al., 1998; Marshall et al., 2001)	Substitution of the key GC base pair in each FNR II core motif (‘+- pyfiD’ = yfiD-m). Deletion of upstream FNR site (stronger activity under complete anaerobiosis; yfiD-P1)	129	FNR
(9) yfiD-WT				
(10) yfiD-m				
(11) yfiD-P1			78	
(12) adhE	Wei et al. (2009)	–	61	FNR
(13) ldhA	Wei et al. (2009)	–	61	unknown
(14) arcA	Wei et al. (2009)	–	434	FNR
(15) gadB	(Conway, 2022; Sangurdekar et al., 2006)*	–	61	unknown

In this study, we systematically compared the performance of 15 previously published oxygen-responsive promoters. We first conducted a parallel throughput microtiter plate-based screening in combination with the oxygen-independent flavin-based fluorescent protein *Cr*LOV from *Chlamydomonas reinhardtii* (Mukherjee et al., 2015) and afterwards a flow cytometry approach based on GFP expression to obtain a robust characterization of the promoters in the presence and absence of oxygen. We identified the most suitable candidates exhibiting low basal expression under aerobic conditions and variable expression strength under anaerobic conditions.

Subsequently, we tested the six most promising candidates in a realistic application example of dynamic metabolic engineering. We considered a two-stage process in which *E. coli* cells grow quickly under aerobic conditions before oxygen supply is switched off to start fermentative production of a target chemical, here lactate. The intention was to induce enforced ATP wasting in the anaerobic production phase by oxygen-dependent expression of the F_1_-subunit of the ATPase, which catalyzes uncoupled ATP hydrolysis often boosting metabolic activity and yields of fermentation products (Boecker et al., 2019; Koebmann et al., 2002). We demonstrate that five of the six selected oxygen-dependent promoter candidates can be utilized to drive expression of the ATPase to optimal levels in the absence of oxygen while allowing growth performance comparable to the wild type in the presence of oxygen. We then used one of the candidate promoters to optimize a two-stage lactate production process and show that the volumetric productivity can be significantly improved over a one-stage process if ATP turnover is automatically increased upon oxygen depletion.

## 2 Materials and methods

### 2.1 Strains and plasmid construction

Supplementary Data 1 summarizes all primers, plasmids, and *E. coli* strains used in this study. All sequence files of plasmid DNA are available through the Edmond repository [https://doi.org/10.17617/3.BOUWY2]. Plasmid propagation and cloning was performed using *E. coli* NEB 5-alpha competent cells (New England Biolabs (NEB), #C2987U). DNA fragments were amplified by PCR using Q5 Hot Start High Fidelity DNA polymerase (NEB, #M0493L) according to manufacturer's instructions. The Zymo-Parts toolkit developed previously in our group (Behrendt et al., 2022) was used for the construction of all plasmids in this work by introducing high-copy (ColE1 ori), medium-copy (15A), and low-copy (RK2) acceptor vectors for *E. coli* (pZP410, pZP334, and pZP419). Golden Gate cloning was performed using 1 μL of 10 × CutSmart buffer (NEB, #B7204), 1 μL of ATP (10 mM), 1 μL of acceptor plasmid (100 ng/μL), 6 μL of insert amplificate or 1 μL of donor plasmid (100 ng/μL), 0.5 μL of *Sma*I, *Bsa*I-HFv2, or *Bbs*I-HF (NEB, #R0141L, R3733L, #R3539L), and 0.5 μL of T4 DNA ligase (NEB, #M0202L) in a total volume of 10 μL. Assemblies were conducted with 6 cycles of 10 min at 37 °C followed by 10 min at 16 °C and a final incubation for 20 min at 37 °C. 5 μL of the reaction mixture were transformed into 15 μL competent cells via heat shock, and blue-white screening was performed with 0.02 g/L 5-bromo-4-chloro-3-indolyl-β-d-galactopyranoside (X-Gal). Promoter candidate sequences were extracted from previous publications (Table 1), and transcription start sites were identified using RegulonDB 11.0 (Tierrafría et al., 2022).

### 2.2 Media and cultivations

For plasmid and colony propagation, *E. coli* strains were cultivated in LB_0_ media (10 g/L tryptone, 5 g/L yeast extract, 5 g/L NaCl) at 37 °C. Antibiotics were added if necessary to concentrations of 100 μg/mL for ampicillin, 100 μg/mL for spectinomycin, and 50 μg/mL for kanamycin. All growth assays were performed with freshly transformed strains and started by inoculating 5 mL LB_0_ medium with a single colony picked from LB_0_ plates containing 15 g/L agar, followed by growth at 37 °C and 160 rpm for 5–6 h. Precultures were diluted 1:2000 into minimal medium (MM: 4 g/L glucose, 34 mM NaH_2_PO_4_, 64 mM K_2_HPO_4_, 20 mM (NH_4_)_2_SO_4_, 9.52 mM NaHCO_3_, 1 μM Fe(SO_4_)_4_, 300 μM MgSO_4_, 1 μM ZnCl_2_, 10 μM CaCl_2_, adapted from (Tanaka et al., 1967)) and grown overnight at 30 °C and 270 rpm to optical densities at 420 nm (OD_420_) between 1 and 2. After centrifugation at 5000 x*g* for 10 min, cells were washed and resuspended in 30 mL of fresh MM to an OD_420_ of 0.2. Cultivation was conducted with volumes of 10 mL in biological triplicates and performed either aerobically at 37 °C in 100 mL shake flasks with baffles at 270 rpm or anaerobically at 37 °C in 25 mL Schott bottles in an anaerobic chamber (Don Whitley Scientific) with an oxygen-free atmosphere (80% N_2_, 10% CO_2_, 10% H_2_) and stirred. For flow cytometry analysis, samples were taken after 7 h of growth.

To compare one-stage with two-stage fermentations, precultures for the one-stage process were inoculated 1:100 from the LB_0_ culture and cultivated at 37 °C overnight without shaking, but precultures for the two-stage process were treated as described above. Cultivation was performed in MM as described above but with 8 g/L glucose, 2 mM (NH_4_)_2_SO_4_, 68 mM NaH_2_PO_4_, and 128 mM K_2_HPO_4_ to enhance the buffer capacity and to prevent acidification by lactate accumulation. One-stage fermentations were carried out anaerobically as stated above, and two-stage fermentations consisted of an aerobic phase with the conditions described above and then transferred into 25 mL glass bottles and switched to the aforementioned anaerobic conditions after 4 h and 40 min.

### 2.3 Microtiter plate fluorescence screening

To screen for expression levels of the different promoter candidates, single colonies were used to perform cultivations of precultures as described above, but with 250 mg/L yeast extract added to the MM. 1 mL of main culture was transferred into each flower-shaped well of 48-well microtiter plates (MTPs) (pH/DO type 2 (LG1/RF), M2P-MTP-48-BOH2, Beckman Coulter GmbH). Plates were sealed with gas-permeable sealing foil (M2P–F-GP-10, Beckman Coulter GmbH). Using the BioLector Pro device (Beckman Coulter GmbH), both anaerobic and aerobic cultivations were performed. The MTP was placed into an anaerobic chamber, which was sealed with an air-tight lid and incubated with a constant stream of nitrogen for the anaerobic cultivation, but left open and incubated with air for aerobic cultivations. Cultivations were performed for 16 h at 37 °C, with a constant humidity of 85%, a shaking frequency of 800 rpm, and constant recording of backscatter (gain 5), fluorescence (gain 10, using the LED filter module Evoglow, excitation wavelength 450 nm (bandpass: 10 nm)/emission wavelength 500 nm (bandpass: 10 nm), M2P-E-OP-418, Beckman Coulter GmbH), and dissolved oxygen (using the LED filter module RF for dissolved oxygen, excitation wavelength 620 nm (bandpass: 25 nm)/emission wavelength 775 nm (bandpass: 50 nm), M2P-E-OP-428, Beckman Coulter GmbH). Cultivations were carried out in biological triplicates, and fluorescence values were normalized to backscatter values for each time point. For each candidate, the normalized background fluorescence measured during cultivation of the strain bearing the empty vector (EV) was subtracted from the normalized fluorescence.

### 2.4 Flow cytometry analysis

Single colonies were used to carry out cultivations as described above. After 7 h of cultivation, 10 μL of the aerobic cultures and 20 μL of the anaerobic cultures were added to 1.5 mL FACS buffer (10 mM TRIS, 10 mM MgCl_2_) with 100 μg/mL chloramphenicol and 100 μg/mL kanamycin – antibiotics inhibiting protein biosynthesis were added to capture the state of expression when the sample was taken. The samples were then incubated at 37 °C for 1 h shaking at 250 rpm, to allow full maturation of fluorescence proteins. Samples were analyzed with the CyFlow Space flow cytometer (Sysmex). A blue laser (488 nm) was used for excitation of GFP, while fluorescence intensity was detected behind an IBP 527/30 optical filter. For mCherry detection, a green laser (561 nm) was used for excitation and the emission was detected using an optical filter IBP 610/30. To gauge GFP expression levels from the different constructs, the median of fluorescence intensity was determined using Flowing Software 2 (Turku Bioscience) – the population was gated on mCherry fluorescence to separate actual cells from background. Three replicates were cultivated per construct with at least fifty thousand cells analyzed per cultivation.

### 2.5 Quantification of metabolites

Quantification of glucose concentrations was performed with the HK assay kit (Megazyme Ltd.). Lactate and succinate were quantified by reversed phase HPLC using the column Inertsil ODS-3 (RP-18, 5 µm, 100 Å, 250 × 4.6 mm) which was operated at 40 °C with a flow rate of 1.0 mL/min and a running buffer consisting of 0.1 M NH_4_H_2_PO_4_, pH 2.6. The injection volume was 2 μL, and detection was performed using an UV detector.

### 2.6 Calculation of yields and rates

The OD_420_ was monitored to calculate biomass concentrations (g_DW_ L^−1^) using a conversion factor of 0.22. For growth-coupled cultivations, specific uptake and production rates in the exponential phase were calculated as follows:

*r*_M_ = *μ* (*c*_M,e_*– c*_M,s_) / (*c*_X,e_*– c*_X,s_) [mmol g_DW_^−1^ h^−1^]

with the specific growth rate *μ*, the end (*c*_M,e_) and start (*c*_M,s_) concentrations of a metabolite M (mmol L^−1^ glucose, lactate, or succinate), and the end (*c*_X,e_) and start (*c*_X,s_) biomass concentrations (g_DW_ L^−1^).

In case of growth arrest, calculation was performed as follows:

*r*_M_ = (*c*_M,e_*– c*_M,s_) / (*X*_Av_ · Δ*t*) [mmol g_DW_^−1^ h^−1^]

with the average biomass concentration *X*_Av_ (g_DW_ L^−1^), and the length of the time period Δ*t* = *t*_e_ – *t*_s_.

Volumetric productivities were calculated using the following formula:

*q =* (*c*_M,e_*– c*_M,s_) / Δ*t* [mmol L^−1^ h^−1^]

Yield calculation was performed between the first and the last time point of the specified production phases by extracting the slope of a linear regression when plotting Δ*c*_M_ (mmol L^−1^) against Δ*c*_Glc_ (mmol L^−1^) for every time point of sampling. These variables were calculated for each of three replicates, and mean and 95% confidence intervals were calculated from these values.

### 2.7 Statistical analysis

*P*-values were calculated with an unpaired two-sample *t*-test using the Microsoft Office Excel Add-in Analysis ToolPak. To determine whether the effect of the promoter on the maximum normalized fluorescence readout is significant, a one-way ANOVA test was carried out using the Microsoft Office Excel Add-in Analysis ToolPak.

## 3 Results and discussion

### 3.1 Promoter candidates exhibit a wide range of expression levels and dynamic ranges

**Fig. 1:**
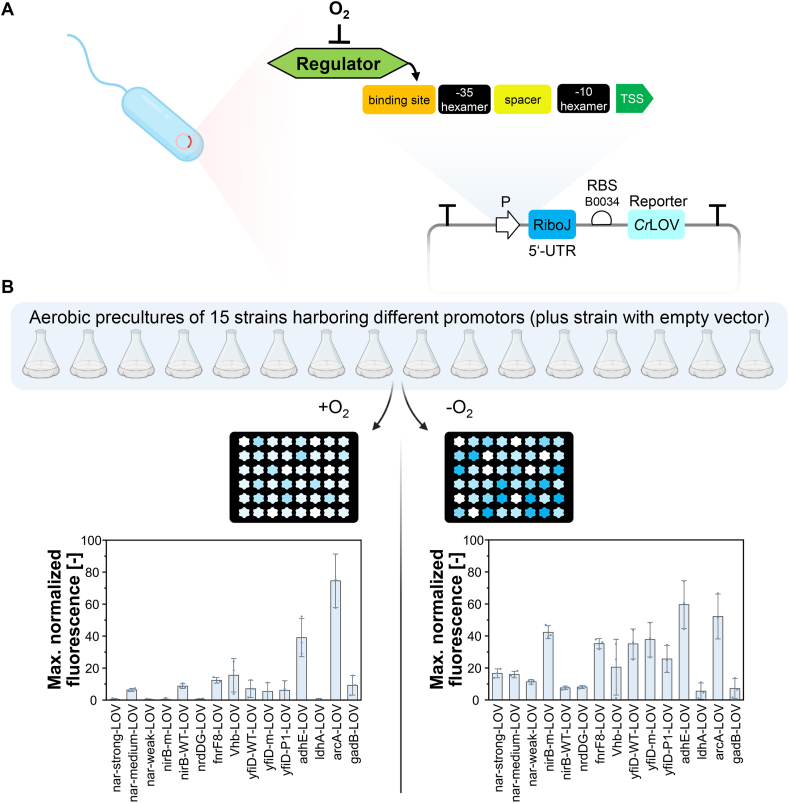
Mechanism of O_2_-dependent promoters (A), and screening principle (B) with maximum normalized *Cr*LOV fluorescence (here illustrated by different intensities of blue colors in the plates) measured during 16 h-cultivation in microtiter plates with (left) and without (right) oxygen. In most cases, the regulator is FNR (see Table 1). In total, one empty vector strain (negative control) and 15 strains harboring the different promoters have been cultivated. Maximum fluorescence values were normalized by dividing by backscatter (biomass) values and by subtracting backscatter-normalized fluorescence values of the negative control. Means (bars) and individual data points (dots) for *n* = 3 biologically independent samples are shown. Error bars indicate 95% confidence intervals. TSS: transcription start site; RBS: ribosome binding sites; UTR: untranslated region; LOV: light, oxygen and voltage sensing protein. Parts of the figure have been created with BioRender.com.

To initially screen for the 15 selected oxygen-dependent promoter candidates (Table 1), we established a parallel throughput workflow using the BioLector Pro cultivation system (Beckman Coulter GmbH), which allows aerobic and anaerobic cultivation in 48-well microtiter plates (MTPs) with constant recording of cell density (backscatter) as well as fluorescence. We chose one of the brightest oxygen-independent fluorescence reporters: the light, oxygen, and voltage (LOV) sensing protein from *Chlamydomonas reinhardtii*, a flavin-based fluorescent protein (Mukherjee et al., 2015). To make its expression solely dependent on the promoter candidate placed upstream of *Cr*LOV and to buffer against genetic context, we utilized strong terminators flanking the entire insert and the ribozyme riboJ upstream of the reporter (Fig. 1A). RiboJ consists of the satellite tobacco ringspot virus (TRSV) ribozyme that cleaves the 5′ untranslated region of the mRNA, leaving identical 5’ ends of mRNAs irrespective of the input promoters (Buzayan et al., 1986; Lou et al., 2012; Moser et al., 2018). Therefore, riboJ acts as an insulator which buffers genetic context effects. Additionally, riboJ was reported to increase expression levels significantly (Clifton et al., 2018), which should increase sensitivity of the assay. As most scenarios of applying oxygen-dependent promoters involve an initial aerobic phase followed by an anaerobic phase in two-stage processes, precultures harboring plasmids with the different promoter candidates were grown aerobically in shake flasks with baffles to maximum cell densities of 0.2–0.5 g/L to guarantee sufficiently high oxygen levels to keep expression low (Moser et al., 2018) (Fig. 1B). Individual strains were then grown aerobically as well as anaerobically (with a constant stream of nitrogen) in the BioLector Pro system, and both culture densities and *Cr*LOV fluorescences of each well were recorded constantly for 16 h. Fluorescence values of the empty vector (EV) control strain were subtracted for each candidate, and the maximum values measured over the whole cultivation period are displayed in Fig. 1B.

A wide range of expression levels could be observed in the pre-screening for the different candidates under both aerobic and anaerobic conditions (Fig. 1B). A substantial increase in the expression level under anaerobic (vs. aerobic) conditions can be seen for most but not all (nirB-WT, arcA, gadB) of the 15 candidates. The oxygen-independent *Cr*LOV reporter is a useful and robust screening tool for promoter characterization enabling a continuous recording of promoter responses to falling oxygen levels *in situ*, however, it has some drawbacks. For example, we monitored biomass formation (backscatter signal) and dissolved oxygen levels that differed between the various candidates during aerobic cultivation in the microtiter plates (Supplementary Fig. 1). Furthermore, fluorescence signals may be distorted by high culture densities reached in the microtiter plates. In line with this, fluorescence signals were found to systematically rise during stationary phase in aerobic cultivations. Therefore, all candidates were additionally subjected to flow cytometry analysis, which should allow more accurate characterization given its dilute measurement setup.

### 3.2 Flow cytometry analysis of promoter candidates

**Fig. 2:**
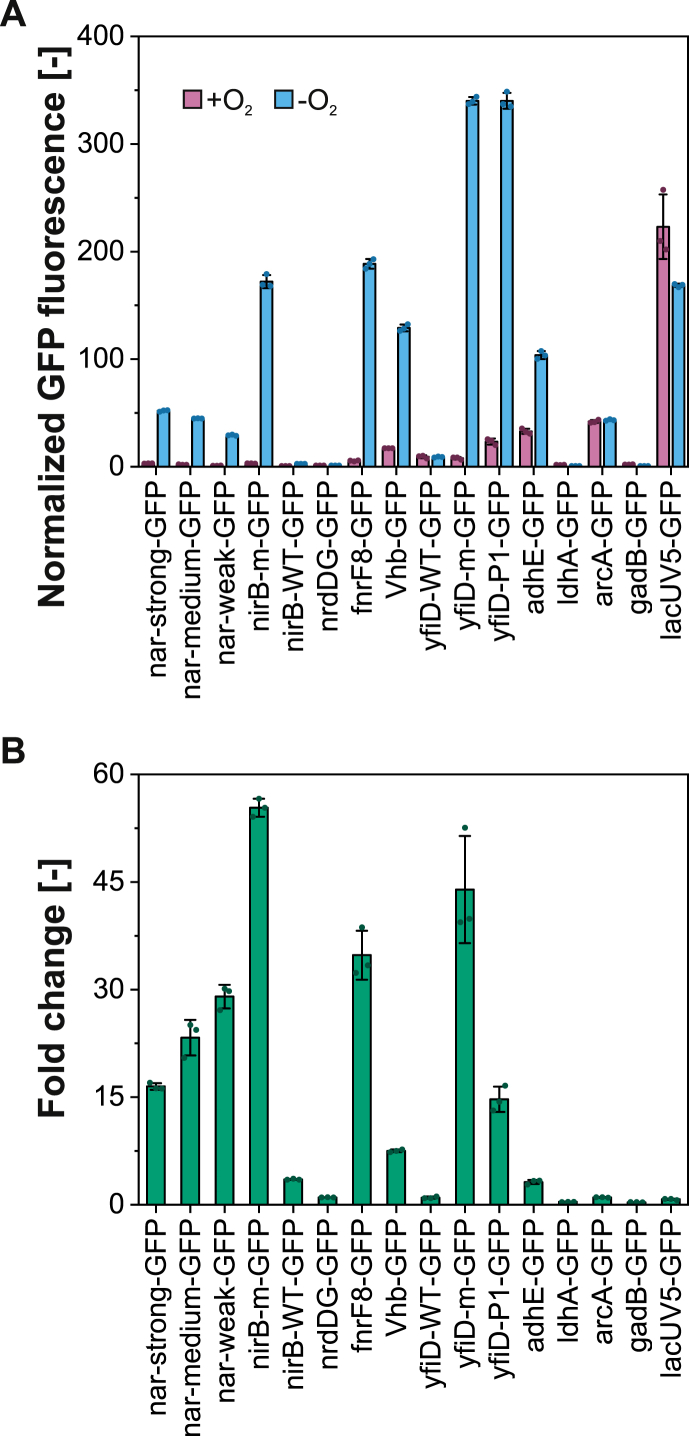
Characterization of oxygen-dependent promoter candidates using flow cytometry. (A) GFP fluorescence was normalized by subtracting fluorescence signals of the empty vector control. (B) Fold changes were calculated by dividing the normalized fluorescence signal obtained under anaerobic conditions by the normalized fluorescence signal obtained under aerobic conditions. Means (bars) and individual data points (dots) for *n* = 3 biologically independent samples are shown. Error bars indicate 95% confidence intervals.

For flow cytometry analysis, all strains harboring the different promoter candidates driving the expression of a GFP reporter were either cultivated aerobically in shake flasks with baffles or in a completely anoxic atmosphere in an anaerobic chamber (following aerobic growth of precultures). The data generated by flow cytometry analysis (Fig. 2) resembled the fluorescence results obtained from the microtiter plate screening, however, lower basal expression levels were observed with flow cytometry during aerobic cultivation. This could be explained with the more dilute measurement setup in flow cytometry and higher background signals in the microtiter plate screening during late cultivation time points with dense cultures (Supplementary Fig. 1). Additionally, the high expression level resulting from the yfiD-WT promoter could not be reproduced during flow cytometry analysis. A reason for this might be the slowly decreasing oxygen concentration during anaerobic cultivation in the BioLector system (Supplementary Fig. 1B). This condition may resemble a microaerobic environment in which the yfiD-WT promoter exhibited higher activity than under complete anaerobiosis (Marshall et al., 2001), which is likely reached faster in the anaerobic chamber used by us prior to flow cytometry analysis. Surprisingly, the previously engineered and characterized nar promoters (nar-strong, nar-medium, and nar-weak) exhibited only small (but significant) differences in strength among each other (one-way ANOVA, *P* = 0.05). The nirB-m, fnrF8, and the yfiD-m and yfiD-P1 promoters demonstrated much higher fluorescence and were similar in strength to the relatively strong constitutive lacUV5 promoter or even stronger (Fig. 2A). Six candidates (nar-strong, nar-medium, nar-weak, nirB-m, fnrF8, yfiD-m) exhibiting variable expression under anaerobiosis but very low signals in the presence of oxygen (Fig. 2B), i.e. a wide dynamic range (fold change), were further tested in a concrete metabolic engineering application (see below). All other candidates were not further considered, as they either were too weak (e.g. nrdDG, ldhA, gadB, yfiD-WT) or exhibited moderate or high basal expression in the presence of oxygen (e.g. Vhb, yfiD-P1, adhE, arcA). Promoter tightness under aerobic conditions is an essential feature for realistic biotechnological applications to prevent unfavorable activities in the aerobic (growth) phase.

### 3.3 Using O_2_-dependent promoter candidates for anaerobic induction of enforced ATP wasting

Next, we aimed to demonstrate that the six most promising promoter candidates we identified are suitable for metabolic engineering applications. As a realistic example application, we consider a two-stage process with aerobic growth phase and anaerobic production phase, where we aim to boost the latter by enforced ATP wasting. As has been shown in several recent studies, increasing the turnover of ATP can be an effective metabolic intervention to optimize the microbial production of target compounds if synthesis of these chemicals is coupled with net formation of ATP (Boecker et al., 2019, 2021a; Chao and Liao, 1994; Hädicke et al., 2015; Liu et al., 2016; Zahoor et al., 2020). One efficient mechanism to induce uncoupled ATP hydrolysis is the expression of the cytosolic F_1_-subunit of the F_O_F_1_-ATPase from *E. coli* (Holm et al., 2010; Koebmann et al., 2002; Liu et al., 2016). ATP wasting typically raises the specific glucose uptake as well as the production rate or/and yield of the desired compound (Boecker et al., 2019, 2021a; Chao and Liao, 1994; Hädicke et al., 2015; Zahoor et al., 2020), but, as a trade-off, typically also reduces the growth rate. As was shown in theoretical (Espinel-Ríos et al., 2022; Klamt et al., 2018) as well as in experimental studies (Boecker et al., 2019, 2021a, 2021b; Zahoor et al., 2020), ATP wasting is particularly efficient to enhance product synthesis in the (growth-arrested) production phase of two-stage processes. However, substrate uptake and productivity were found to follow a biphasic response curve when increasing ATPase levels, with an optimum reached at medium ATPase activities (Boecker et al., 2021b). Hence, effective utilization of the enforced ATP wasting strategy requires timely activation of the ATPase (here in the anaerobic production phase) at a suitable level.

**Fig. 3:**
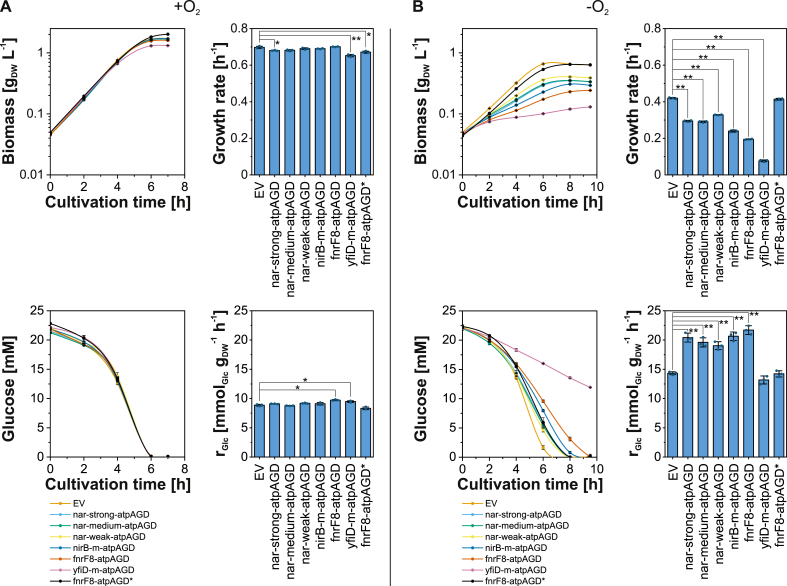
Time courses of biomass and glucose concentrations as well as specific growth rates and specific glucose uptake rates (r_Glc_) of *E. coli* strains expressing the cytosolic F_1_-subunit of the ATPase from *E. coli* under transcriptional control of six oxygen-dependent promoter candidates under aerobic (A) and anaerobic (B) conditions. An inactive ATPase with a G152R point mutation in the (catalytically active) β-subunit (Lee et al., 1991) was tested additionally to the empty vector (EV) control in combination with the fnrF8 promoter (fnrF8-atpAGD*). Means (and individual data points (dots) in the bar diagrams) for *n* = 3 biologically independent samples are shown. Error bars indicate 95% confidence intervals. Statistically significant differences with *P*-values <0.01 and 0.001 are indicated by bars with single (*) and double (**) asterisks as assessed by an unpaired two-sample *t*-test.

Therefore, in a first step, we sought to adjust an optimal level of ATPase expression with the selected promoter candidates using established genetic tools. In a realistic application scenario, this optimum would have to be reached briefly after switching the culture from a completely aerobic to an anaerobic state. Accordingly, we tested the effects of ATPase expression driven by the candidates by growing precultures in completely aerobic conditions, to then split these into aerobic and anaerobic main cultures (Fig. 3A&B). In order to slowly approach (and not to exceed) the correct ATPase level, its expression was driven from a low-copy broad-host range (RK2 replicon) vector (Blatny et al., 1997a, 1997b) in the *E. coli* K-12 MG1655 wild-type strain. Typically, successful expression of the ATPase to optimal levels in the wild-type strain would result in enhanced specific glucose uptake rates and reduced growth rates, which we used as indicators for tightness under aerobic conditions and performance under anaerobic conditions.

As expected and desired, growth (μ ≈ 0.7 h^−1^) and specific glucose uptake (r_Glc_ ≈ 9–10 mmol_Glc_ g_DW_^−1^ h^−1^) proceeded nearly identically under aerobic conditions when comparing the different promoter candidates to the EV control strain (Fig. 3A). Only the strong fnrF8 and yfiD-m promoters exhibited basal expression levels (Fig. 2A) that resulted in marginal (but statistically significantly) elevated uptake rates (Fig. 3A). However, all candidates appear sufficiently tight in the presence of oxygen to guarantee fast growth. In the absence of oxygen, large differences to the EV strain could be observed: growth was significantly slower (μ ≈ 0.08–0.33 h^−1^) compared to the EV strain (μ ≈ 0.42 h^−1^), and specific glucose uptake rates were strongly elevated for nar-strong, nar-medium, nar-weak, nirB-m, and fnrF8 to levels of ∼19–22 mmol_Glc_ g_DW_^−1^ h^−1^ compared to 14.3 mmol_Glc_ g_DW_^−1^ h^−1^ for the EV strain (Fig. 3B). Indeed, a positive correlation between the expression level and specific glucose uptake rate could be observed for these candidates (compare Fig. 2, Fig. 3B). In contrast, a slightly decreased glucose uptake rate (13.2 ± 1.1 mmol_Glc_ g_DW_^−1^ h^−1^) was observed for the yfiD-m promoter. This is likely caused by sub-optimal, i.e. too high ATPase expression levels driven by this candidate (which exhibited the strongest fluorescence signal; see Fig. 2A), a phenomenon related to a biphasic response curve of the glucose uptake rate to increased ATPase expression levels (Boecker et al., 2021b). To the best of our knowledge, with the fnrF8 promoter we have engineered *E. coli* to reach the highest specific glucose uptake rate of 21.7 mmol_Glc_ g_DW_^−1^ h^−1^ reported to date under anaerobic conditions in minimal medium during exponential growth. This represents an increase of 51 % compared to the control strain and of 22.6 % compared to the value reported previously (Boecker et al., 2021b). To confirm that the wasting effect is solely caused by the hydrolyzing activity of the expressed F_1_-subunit of the ATPase, and not (in part) by the higher ATP demand caused by plasmid maintenance and protein expression, we also tested whether abolished ATP hydrolyzing activity caused by a G152R point mutation in the (catalytically active) β-subunit (Lee et al., 1991) resulted in similar patterns as for the wild-type strain with the empty vector. Even when expression of this modified ATPase* was driven by a strong promoter (fnrF8), growth slowed down only very slightly (μ ≈ 0.41 h^−1^), and the specific glucose uptake rate also decreased modestly to 14.2 mmol_Glc_ g^−1^ h^−1^ (Fig. 3B), providing strong evidence that this is not the case.

### 3.4 Optimizing a two-stage lactate production process using an ATPase under dynamic control of the nirB-m promoter

We finally applied one of the developed dynamic ATPase expression systems for the efficient production of a specific target metabolite, d-lactate, by *E. coli*. As was shown in (Hädicke et al., 2015), specific glucose uptake and lactate production rates as well as lactate yield of a designed *E. coli* lactate producer strain can be enhanced by enforced ATP wasting since generation of lactate from glucose is coupled to net synthesis of ATP. However, due to the significantly reduced growth rate, biomass accumulated only slowly, leading to a lower volumetric productivity compared to the strain without ATP wasting. This calls for a two-stage process with decoupled aerobic growth and anaerobic lactate production phase, in which the ATPase is induced in the second phase to boost lactate synthesis. It should be noted that, even with a two-stage process, there remains a trade-off between volumetric productivity and product yield: higher accumulation of biomass in the growth phase (i.e., a later switch to the production phase) may increase the volumetric productivity of the entire process but will simultaneously reduce product yield since less substrate is available for product synthesis. Typically, and this was also considered in our case study, the goal is to maximize the productivity under the constraint of a reasonable (possibly slightly reduced) product yield.

**Fig. 4:**
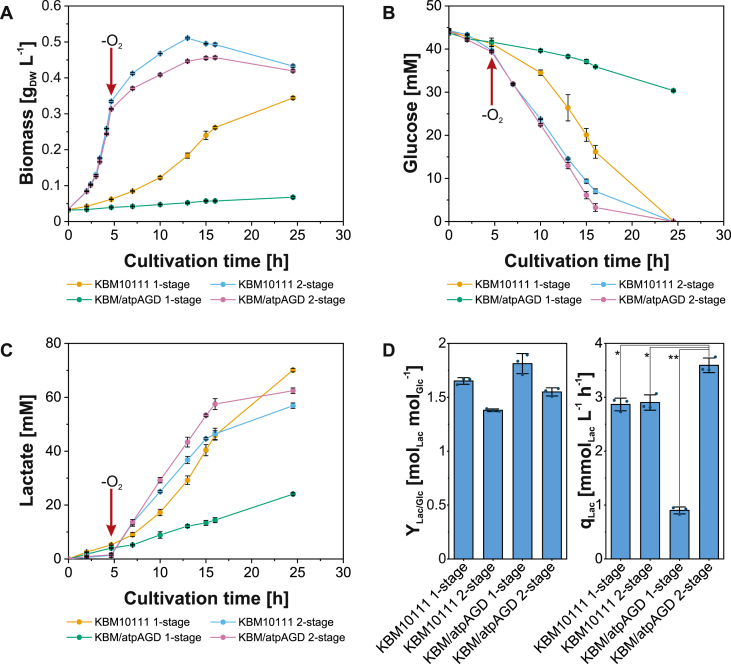
One-stage vs. two-stage process for lactate production by the original lactate producer strain KBM10111 and the KBM/atpAGD strain (KBM10111 with pZP1036-nirB-m-atpAGD_RK2). (A) Growth curves with the time point of switch from aerobic to anaerobic conditions (for the two-stage fermentations) indicated by the red arrow. (B) Consumption of glucose. (C) Lactate production. (D) Lactate yields from glucose (Y_Lac/Glc_) and volumetric productivities (q_Lac_) of the first 16 h for the different setups. Means and individual data points (dots in panel D) for *n* = 3 biologically independent samples are shown. Error bars indicate 95% confidence intervals. Statistically significant differences with *P*-values <0.01 and 0.001 are indicated by bars with single (*) and double (**) asterisks as assessed by an unpaired two-sample *t*-test.

We chose the nirB-m promoter to dynamically drive ATPase expression due to its moderate strength combined with a wide dynamic range compared to the other candidates (Fig. 2). Although the stronger fnrF8 promoter resulted in higher specific glucose uptake rates in the wild type strain than with nirB-m (Fig. 3B), we opted against this promoter because its high ATPase expression levels could lead to adverse effects in the KBM10111 (= MG1655 *ΔadhE::cat ΔackA-pta*) background strain used herein (Hädicke et al., 2015). In this lactate producer strain, excretion of the other main fermentation products ethanol and acetate is suppressed, which likely reduces its metabolic flexibility and robustness. As *E. coli* naturally excretes lactate as a fermentative product under anaerobic conditions, it was not necessary to artificially induce a production pathway for lactate in this case. For a realistic process scenario, we compared the performance of this original production strain KBM10111, lacking any plasmid (and consequently any additional metabolic or growth burden; Fig. 4) against the same strain bearing the dynamic expression system with the nirB-m promoter and atpAGD genes (strain KBM10111 with pZP1036-nirB-m-atpAGD_RK2, in the following abbreviated as ‘KBM/atpAGD’; Fig. 4). Two operational modes were assessed for both strains: one-stage fermentation proceeding only under anaerobic conditions, and two-stage fermentation with an aerobic growth phase followed by an anaerobic production phase, in which further cell growth was prevented by nitrogen limitation. Both setups were started with identical initial cell densities (Fig. 4A). The time point to switch from aerobic to anaerobic conditions was chosen such that most of the nitrogen present in the medium would be consumed, and the remaining fraction of nitrogen would be sufficient to allow the cell to adapt the proteome to the anaerobic state and to induce ATPase synthesis.

This state was reached after 4:40 h (indicated by the red arrow in Fig. 4A). Until then, aerobic growth for both the KBM10111 (μ ≈ 0.52 h^−1^) and KBM/atpAGD strain (μ ≈ 0.50 h^−1^) proceeded in a similar exponential manner, with a slight advantage for the KBM10111 strain, which may be explained by the burden caused by plasmid maintenance in the KBM/atpAGD strain. After switching to the anaerobic phase of both two-stage processes, optical densities still increased for a while at a slow pace, indicating that some nitrogen was still present at the switching time point as intended. Regarding the anaerobic one-stage process for both strains, as expected, the difference in specific growth rates was again much higher since growth was perturbed strongly by the permanent ATPase expression in the KBM/atpAGD strain (μ ≈ 0.04 h^−1^, KBM10111: μ ≈ 0.13 h^−1^). Glucose and lactate concentrations were continuously recorded for all set-ups to evaluate the effect of dynamically induced ATPase expression using the nirB-m promoter on productivities and yields. In fact, both of these essential performance parameters could be optimized by our strategy: while the KBM10111 strain was similarly productive in the first 16 h in both the one-stage and two-stage mode (q_Lactate_ ≈ 2.9 mmol_Lac_ L^−1^ h^−1^), the KBM/atpAGD strain performed significantly better (+24%) in the 2-stage process (q_Lactate_ ≈ 3.6 mmol_Lac_ L^−1^ h^−1^) (Fig. 4D). Consequently, over the entire process, glucose was taken up and lactate produced most rapidly in this setup (Fig. 4B). Additionally, the product yield could be improved by nirB-m-driven ATPase expression both for the one-stage process (+10%) as well as for its two-stage counterpart (+12%) compared to the respective KBM10111 cultivations, although the dynamically regulated two-stage mode had a slightly lower final product titer (Fig. 4C) and product yield (Fig. 4D) than the KBM10111 strain in one-stage mode. This remaining minor trade-off between productivity and product yield (partially already discussed above) can be explained by the very low lactate yield during aerobic growth of the two-stage process compared to the growth-coupled production of lactate in the one-stage process.

## 4 Conclusions

In this study, we conducted a systematic characterization of oxygen-dependent promoters in *E. coli* and investigated their suitability for dynamic metabolic engineering applications. Previously, only single candidates or their modified versions had been analyzed, and only few of these had been utilized in concrete applications. Our approach of using two screening methods with different fluorescent reporters aimed at filling this gap by providing a comprehensive overview of promoter strengths and dynamic ranges. We found large differences among the various promoter candidates, especially with respect to their basal activity under aerobic conditions. As demonstrated in this study, this dataset facilitated the selection of suitable candidates for oxygen-dependent dynamic regulation of gene expression for bioprocess design. By combining these candidates with other available genetic tools such as ribozymes, RBSs, terminators, or vectors of various copy numbers, the expression level can be fine-tuned as desired. Consequently, this will allow application of these promoters in dynamic metabolic engineering approaches that require a narrow window of gene expression to be functional. One example demonstrated in this work is the optimization of the productivity of a two-stage production process, here with lactate as target product, by dynamically enforcing ATP wasting using the nirB-m promoter to boost the metabolic activity of growth-arrested cells in the anaerobic production phase. Compared to the strain without dynamically induced ATP wasting, the volumetric productivity could be increased by 24 %. Other possible applications include the induction of entire product pathways when switching to anaerobic conditions. Taken together, we believe that our work and results will be valuable in bringing dynamic metabolic engineering and bioprocess design concepts that use oxygen tension as a trigger closer to realistic applications.

## Author statement

**Julian Wichmann**: Conceptualization, Methodology, Investigation, Data Curation, Validation, Writing – Original Draft, Writing - Review & Editing. Visualization. **Gerrich Behrendt**: Methodology, Investigation, Data Curation, Validation, Writing - Review & Editing**. Simon Boecker**: Conceptualization, Investigation, Validation, Writing - Review & Editing. Visualization. **Steffen Klamt**: Conceptualization, Writing – Original Draft, Writing - Review & Editing, Supervision, Project Administration, Funding Acquisition.

## Declaration of competing interest

The authors declare that they have no competing interests.

## Data Availability

Data are available in the Supplementary data file and on https://doi.org/10.17617/3.BOUWY2
