# Supplementary material for: Characterizing and utilizing oxygen-dependent promoters for efficient dynamic metabolic engineering
Source: Metab Eng. 2023 May;77:199–207. doi: 10.1016/j.ymben.2023.04.006 (PMC10208419; doi:10.1016/j.ymben.2023.04.006)
Supplement: Multimedia component 1 [file mmc1.pdf]

Supplementary Figures

Characterizing and utilizing oxygen-dependent promoters for efficient dynamic metabolic engineering

Julian Wichmann[1], Gerrich Behrendt[1], Simon Boecker[1], and Steffen Klamt[1,*]

[1)] Analysis and Redesign of Biological Networks, Max Planck Institute for Dynamics of Complex Technical Systems, Sandtorstr. 1, 39106 Magdeburg, Germany

[*)] Corresponding author: klamt@mpi-magdeburg.mpg.de

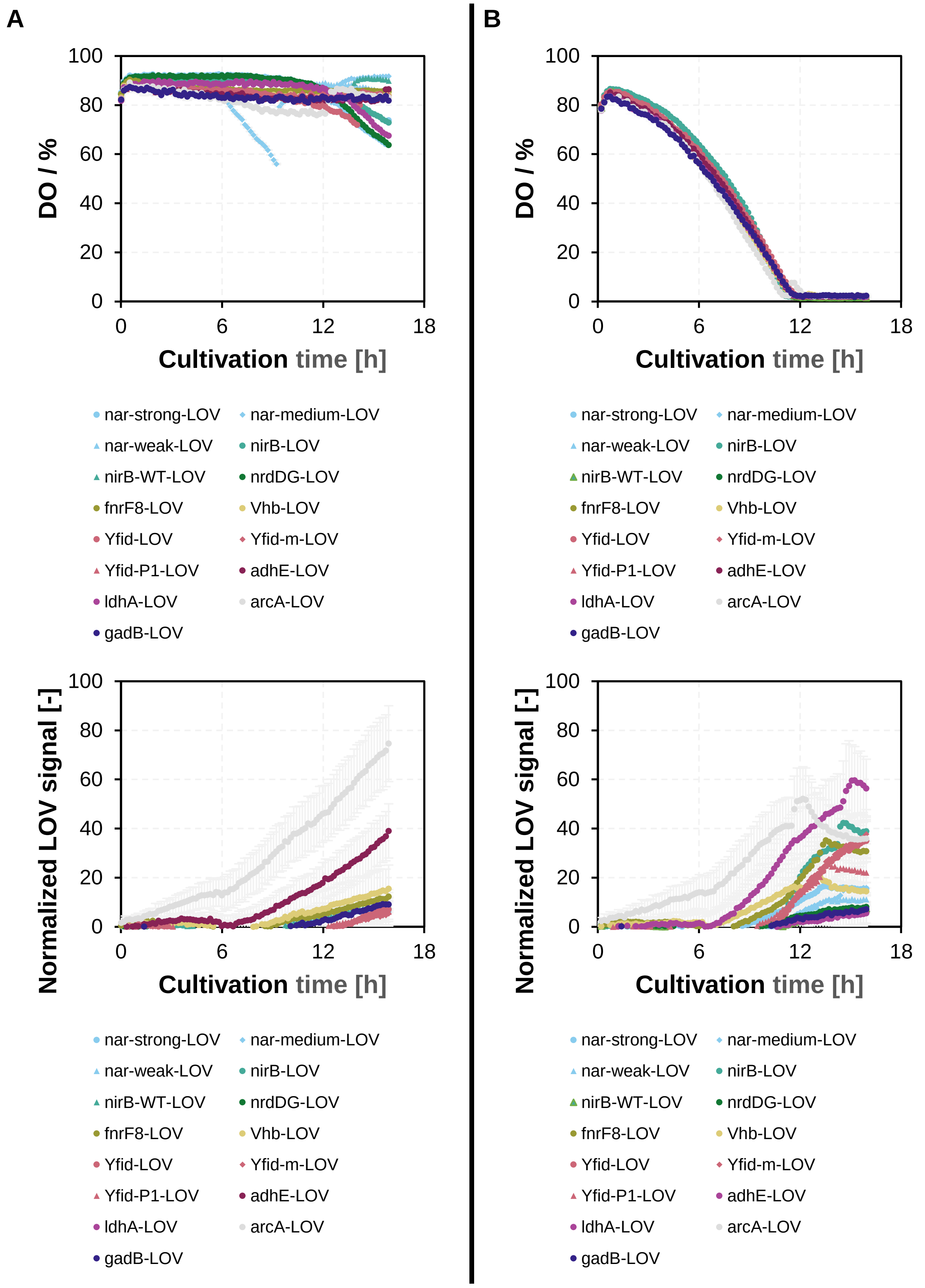

Supplementary Figure 1. Dissolved oxygen content (DO) in the culture medium and normalized fluorescence signals of the LOV reporter during aerobic (A) and anaerobic (B) cultivation in microtiter plates in the BioLector Pro system (Beckman Coulter GmbH).
